# The Treatment of Fractures from a Common-Sense Point of View

**Published:** 1907-11-23

**Authors:** 


### Points in Surgery.

### THE TREATMENT OF FRACTURES FROM A COMMON-SENSE POINT OF VIEW.

### XIII. FRACTURE OF THE PATELLA.

Fractures of the patella may be stellate or transverse. The stellate variety is much the rarer of the two; in fact, one does not often come across examples of this injury even in a great hospital. When it does occur, it is produced by direct violence applied to the point of the knee, as the result of which the patella is smashed into a number of small pieces which radiate from the point struck, just as a sheet of plate-glass splinters when it is broken. There is generally little or no displacement, because the aponeurosis of the quadriceps extensor remains intact and the fragments are thus kept in position. The diagnosis is obvious. If the nature of the injury is by any chance overlooked, and the patient allowed to attempt to walk, the consequences may be awkward, because in his attempts to extend the leg the patient may tear the aponeurosis and add a new complication to the condition of affairs. Treatment resolves itself into keeping the limb at rest to allow the effusion into the knee-joint, which is nearly always severe, to subside and to give the fragments a chance of uniting. The effusion tends to go on spontaneously in the course of time, but the process may be hastened by applying an evaporating lotion to the joint. In severe cases, however, it may be necessary to aspirate the joint and remove the excess of fluid. The limb should be kept at rest on a back splint which is raised on a patellar junk, the object of this being to keep the quadriceps extensor in a position of relaxation. It is useless to attempt to wire the fragments in this injury, because their number is great, and the size of each individual fragment is small. Union is fairly satisfactory in most cases, because the fragments are kept in apposition by the aponeurosis. Limitation of movement at the knee-joint is sometimes caused by subsequent irregularity in the line of the articular surface, and osteo-arthritis has been said to be a constant sequela, but there is no proof of this.

Transverse fracture of the patella is always due to indirect violence. What usually happens is that the patient slips when going upstairs, and contracts his quadriceps extensor violently in the effort to recover himself. At this moment the knee is flexed, and the patella is broken in two across the lower end of the femur by the pull of the muscle. When the patella gives way the muscle goes on contracting, so that the aponeurosis is torn also, a fact which accounts for the wide separation of the fragments which is seen in this condition. The diagnosis is obvious in view of the patient's history, his inability to extend his leg, and the presence of a depression between the two fragments in front of the knee.

#### Treatment.

The surgeon has the choice of treating the condition by operation or by retentive apparatus. There can be no doubt that operative treatment is the ideal one. If the case is treated by retentive apparatus, fibrous union is the most favourable result that can possibly be hoped for; and fibrous union means loss of power in the limb, with inability to extend it completely because of the lengthening of the quadriceps extensor muscle. On the other hand the operation is one not without danger, on account of the risk of introducing sepsis into the synovial cavity. The synovium does not appear to have the same resistance to infection that the peritoneum has, and the results of septic arthritis in a large joint like the knee are most serious. It may even cost the patient his life. But if the operation is performed with strict asepsis, such a complication should be avoidable. Most surgeons nowadays use indiarubber gloves for this, if for no other operation; for there can be no doubt that they can more certainly be made sterile than can the hands. In the absence, therefore, of any constitutional contra-indication, such as extreme old age or albuminuria, the right treatment is to operate.

How soon after the accident should the operation be performed? Some surgeons do it almost at once, but it is better to wait three or four days, during which time the limb may be made thoroughly aseptic with preparatory dressings; the effusion in the joint subsides, and the muscular spasm disappears. There is no indication to wait longer than this, since each day's delay retards the patient's ultimate recovery.

The best incision to employ is a curved one, convex outwards. It is better than a median vertical incision, because the ends of the wire do not lie underneath it, as they do if a median incision is used. The joint is washed out until all blood-clot is removed; this is important because there is no better nidus for pyogenic micro-organisms than old clot. The fragments of bone are drilled in a manner identical with that described under " Fracture of the Olecranon," and are brought together with a piece of silver wire whose ends are twisted, cut short, and hammered down. The operation is comparatively easy if the two fragments are of approximately the same size. But when the patella gives way near its apex and the lower fragment is small, it may be difficult to introduce the wire without splitting the bone. In this case it may be found easier to unite the fragments with a piece of stout silk. The wound is closed with interrupted sutures, and the limb put up on a back splint. The dressings should be renewed daily for the first three or four days. In some cases there is a yellowish discharge without any rise in the temperature. It may be mistaken for pus, but it is really blood-clot which has been altered by the synovial fluid, and need not give cause for alarm. Passive movement should be begun early, and the patient may begin to get about at the seventh week.

If it is decided to adopt non-operative treatment, a satisfactory method is the one known as "the Middlesex method." The limb is put up on a back splint until the effusion has subsided. The lower fragment is then fixed by transverse pieces of strapping, which are firmly applied round the joint just below it. The upper fragment is brought down into apposition by strapping over it a piece of elephant plaster, which is cut away over the patella; to the sides of the elephant plaster pieces of elastic tubing are attached, which are stretched and fixed to the foot. The two fragments are thus kept more or less in apposition. The apparatus must be retained for from six to eight weeks.

